# Rare antigen‐negative red blood cells from pluripotent stem cells for precision transfusion medicine

**DOI:** 10.1111/trf.70243

**Published:** 2026-04-24

**Authors:** Naomi Gunawardena, Hyun Hyung An, Randall W. Veliquette, Edmund Carvalho, Sambit Dalui, Kaoru Takasaki, Eric Wafula, Deborah L. French, Giulia Pavani, Stella T. Chou

**Affiliations:** ^1^ Division of Hematology, Department of Pediatrics Children's Hospital of Philadelphia Philadelphia Pennsylvania USA; ^2^ Division of Transfusion Medicine, Department of Pathology and Laboratory Medicine Children's Hospital of Philadelphia Philadelphia Pennsylvania USA; ^3^ Department of Cell and Molecular Biology Perelman School of Medicine Philadelphia Pennsylvania USA; ^4^ Immunohematology and Genomics Laboratory New York Blood Center Enterprises Rye New York USA; ^5^ Perelman School of Medicine Philadelphia Pennsylvania USA; ^6^ Department of Biomedical and Health Informatics Children's Hospital of Philadelphia Philadelphia Pennsylvania USA; ^7^ Center for Cellular and Molecular Therapeutics Children's Hospital of Philadelphia Philadelphia Pennsylvania USA; ^8^ Division of Experimental Pathology, Department of Pathology and Laboratory Medicine Children's Hospital of Philadelphia Philadelphia Pennsylvania USA

**Keywords:** alloimmunization, induced pluripotent stem cells, MAM antigen, rare red blood cells

## Abstract

**Background:**

Blood bank identification of antibodies against high‐prevalence antigens remains a challenge due to the scarcity of antigen‐negative reagent red cells sourced from blood donors. The MAM antigen, encoded by *EMP3*, is one such antigen associated with red cell alloimmunization and hemolytic disease of the fetus and newborn.

**Study Design and Methods:**

We used CRISPR‐Cas9 gene editing to generate an *EMP3* knockout (EMP3KO) induced pluripotent stem cell (iPSC) line from a type O, Rh null parent line, enabling production of rare MAM‐negative red blood cells. Since a prior study suggested that loss of EMP3 may enhance erythroid proliferation, we hypothesized that EMP3KO could both yield a rare reagent cell and potentially improve erythroid expansion to support scalable production. Transcriptomic analysis allowed us to further investigate the effect of *EMP3* loss in late erythroblasts.

**Results:**

EMP3KO cells differentiated efficiently into erythroid cells, showing >95% CD235/CD71 co‐expression and orthochromatic erythroblast morphology. Compared to unedited cells, no proliferative advantage was observed, contrasting with prior non‐isogenic cell models. Agglutination assays confirmed complete loss of MAM antigen and demonstrated the diagnostic utility for identifying MAM antibodies. Transcriptomic profiling of EMP3KO erythroblasts revealed expression of key erythroid genes, as well as regulators of proliferation and heme metabolism, was comparable to the parent line.

**Discussion:**

This study demonstrates that iPSC technology combined with gene editing can generate rare antigen‐negative RBCs for immunohematology applications. Beyond MAM, this platform offers a strategy to create additional rare RBC phenotypes, advancing precision transfusion medicine and improving antibody identification against high‐prevalence antigens.

**Abbreviations**
BFU‐E: burst forming unit‐erythroid
CFU‐E: colony forming unit‐erythroid
DGE: differential gene expression
EMP3: epithelial membrane protein 3
gRNA: guide RNA
GSEA: gene set enrichment analysis
HDFN: hemolytic disease of the fetus and newborn
HPC: hematopoietic progenitor cell
iPSC: induced pluripotent stem cell
iRBC: induced pluripotent stem cell‐derived red blood cell
KO: knockout
PBS: phosphate buffered solution
PCA: principal component analysis
RBC: red blood cell

## 1 INTRODUCTION

Alloantibodies against red blood cell (RBC) antigens can form upon exposure to incompatible RBCs, leading to antibody‐mediated destruction of cells that express these antigens upon repeat exposure.^1^ In hemolytic disease of the fetus and newborn (HDFN), maternal alloantibodies are formed against incompatible fetal RBC antigens, leading to immune‐mediated destruction of fetal and neonatal cells.^2^ Maternal sensitization to RhD is the most common cause^3^ and is readily diagnosed and managed with RhD‐negative RBCs.^4^ In contrast, alloimmunization and HDFN caused by immunization to high‐prevalence RBC antigens pose significant diagnostic and therapeutic challenges.^5^, ^6^ Since these antigens are present on nearly all donor and reagent red cells, antibody identification panels often yield pan‐reactivity, resulting in the inability to determine the antibody specificity and select appropriate donor units for safe transfusion. Diagnosis requires rare reagent cells combined with molecular methods limited to reference laboratories. The scarcity of appropriate red cells for antibody identification delays care and increases patient morbidity and mortality.

The MAM blood group system comprises a single high‐prevalence antigen,^7^ encoded by the epithelial membrane protein 3 (*EMP3*) gene.^8^ EMP3 is a transmembrane signaling protein with context‐dependent oncogenic and tumor‐suppressor roles in various cancers.^9^ In hematopoiesis, MAM‐negative CD34^+^ hematopoietic progenitor cells (HPCs) exhibit increased erythroid proliferation in vitro but MAM‐negative individuals do not demonstrate polycythemia.^8^ The MAM antigen is expressed on epithelium and RBCs of nearly all individuals,^7^, ^8^ and was recognized as a distinct blood group by the International Society of Blood Transfusion after antibodies were identified in the plasma of three pregnant women, with “MAM” representing the proband's initials.^7^ Alloanti‐MAM can cause HDFN and hemolytic transfusion reactions, but diagnosis is extremely challenging due to the limited availability of MAM‐negative reagent cells.^7^ This scarcity of MAM‐negative RBC products also limits compatible transfusion support.

We and others have demonstrated that human induced pluripotent stem cells (iPSCs) can be used to generate iPSC‐derived RBCs (iRBCs) with precisely defined antigen profiles, including Rh null and U‐negative RBCs.^10^, ^11^, ^12^ This platform provides a renewable and customizable source of rare phenotype cells for diagnostic testing and potentially future therapeutic applications.^10^, ^13^ Building on our previously described type O, Rh null iPSC model,^10^ we generated EMP3‐null iPSCs to produce MAM‐negative iRBCs. Comparative analysis of the isogenic control and MAM‐negative iRBCs showed similar erythroid maturation and proliferation, and the diagnostic utility of MAM‐negative iRBCs was demonstrated using patient plasma containing anti‐MAM antibodies.

## 2 METHODS

### 2.1 iPSC gene targeting and hematopoietic differentiation

**FIGURE 1:**
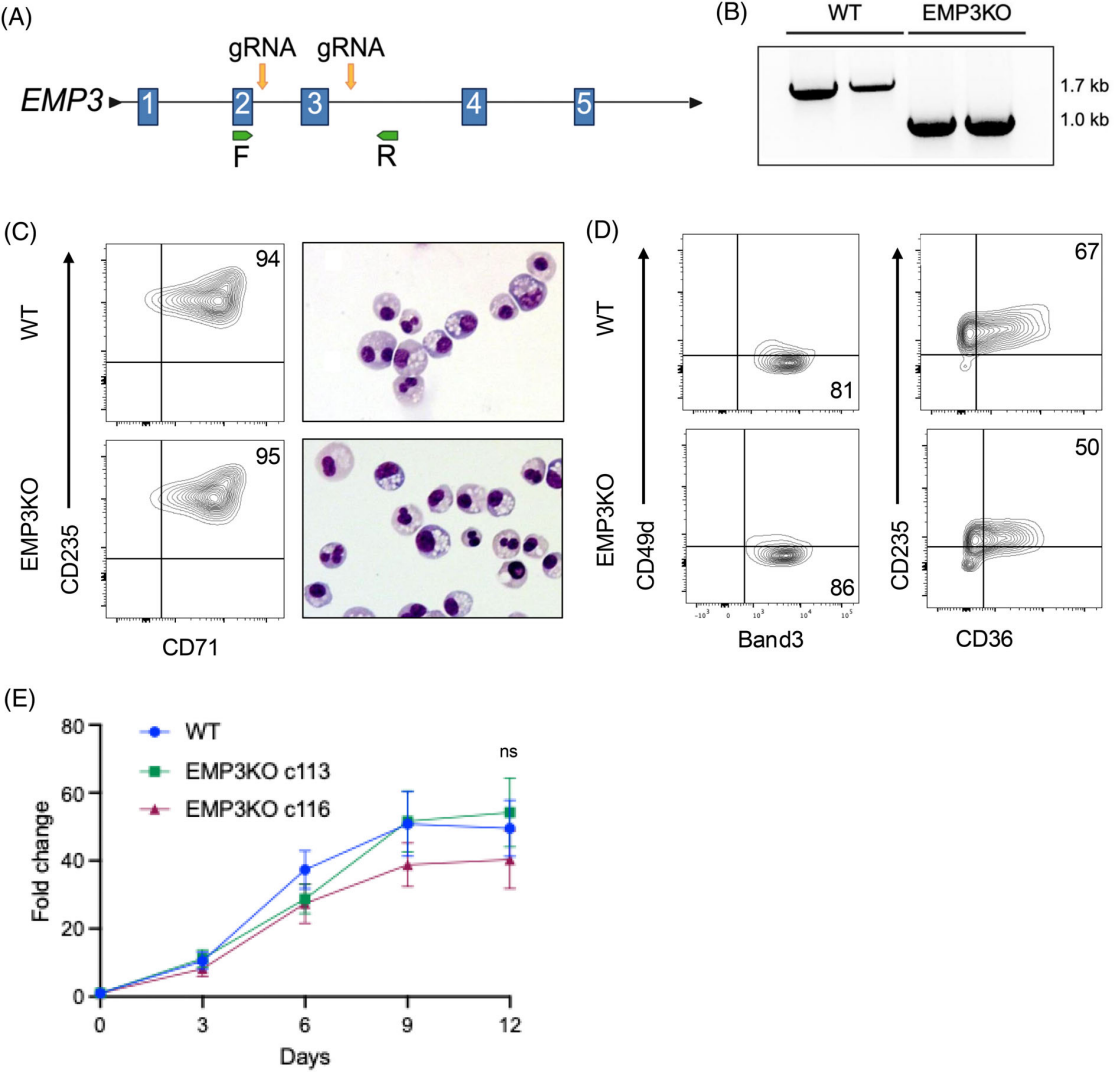
EMP3 knockout and iRBC generation. (A) Schematic of CRISPR‐Cas9‐mediated deletion of *EMP3* exon 3 in an Rh null iPSC line. (B) PCR products amplified with primers targeting exon 2 and intron 3 (green arrows in 1A) identify iPSC clones with a deletion of ~750 bps. (C) Representative flow cytometric analysis of cell surface erythroid maturation markers and morphology (May‐Grunwald Giemsa stain) of day 12 WT and EMP3KO iRBCs. (D) Representative flow cytometric analysis of erythroid maturation surface antigens CD49d, Band3, and CD36 in day 12 WT and EMP3KO iRBCs. (E) Fold expansion of iRBC per HPC in erythroid culture on days 3, 6, 9, and 12 (*n* = 8 independent assays; ns, non‐significant).

The *EMP3* knockout (EMP3KO) line was generated in an established Rh null iPSC line (WT17.6.36) using the CRISPR‐Cas9 nuclease system, following our described protocol.^14^ The Rh null cells were selected in part based on the hypothesis that EMP3 disruption could enhance iRBC proliferation and facilitate scaled production. Guide RNAs (gRNAs) were designed to target *EMP3* exon 3 to introduce a frameshift and disrupt protein expression (Figure 1A). All resulting iPSCs demonstrated normal copy number variation profiles. gRNAs and PCR primer sequences are provided in Table S1. iPSCs were differentiated by embryoid body formation to produce primitive hematopoietic progenitor cells (HPCs, CD41^+^CD235^+^) and subsequently cultured in erythroid differentiation media to produce iRBCs as previously described^10^, ^15^ and detailed in Supporting Information.

### 2.2 Tube agglutination assay

Patient plasma samples containing anti‐D, anti‐MAM, or inert control plasma were obtained from a rare frozen sera collection at the New York Blood Center or from study subjects under a protocol approved by the Children's Hospital of Philadelphia Institutional Review Board. iRBCs were incubated with plasma samples or commercial monoclonal IgM typing reagents anti‐k and anti‐K (Werfen, Norcross, GA) and low ionic strength saline enhancement solution (Quidel Ortho, Raritan NJ) at 37°C for 30 min, followed by centrifugation at 3000 rpm for 1 min. Agglutination was not assessed at immediate spin. Cells were washed with phosphate buffered saline (PBS) and 2 drops of anti‐IgG (Werfen) were added. Following centrifugation, the cell button was gently resuspended, and macroscopic agglutination was assessed visually.

### 2.3 Gene expression analysis of wild‐type and EMP3KO iRBCs

Total RNA was isolated from day 12 erythroblasts generated from wild‐type (WT; clones 36 and 6) and EMP3KO (clones 113 and 116) iPSCs (7 biological replicates/genotype). Bulk RNA sequencing was performed on the NextSeq 1000 Sequencing System (Illumina). Detailed data quality control and analysis methods are in Supporting Information. Briefly, we performed differential gene expression (DGE) and gene set enrichment analysis (GSEA) using MSigDB Hallmark pathways. Separate analyses were conducted to examine specific subsets of genes related to erythroid differentiation including gene sets identified by Li et al.^16^ that are up‐ or downregulated during the transition from erythroid burst forming unit (BFU‐E) to colony forming unit (CFU‐E) and from CFU‐E to proerythroblast, along with known transcription factors involved in these transitions.

## 3 RESULTS

### 3.1 EMP3 knockout iPSCs and generation of MAM‐negative iRBCs

An EMP3KO iPSC line was generated from our established Rh null iPSC line.^10^ CRISPR‐Cas9 editing was used to delete a 750 bp fragment encompassing *EMP3* exon 3 (Figure 1A). Clones with homozygous deletions were identified by a ~1.0 kilobase (kb) PCR amplicon, compared to a 1.7 kb WT product (Figure 1B), and cDNA sequencing confirmed a premature stop codon in the EMP3KO clones (Figure S1).

Using our established erythroid differentiation protocol that recapitulates primitive (embryonic) hematopoiesis,^10^ both WT and EMP3KO day 12 iRBCs demonstrated ~95% co‐expression of erythroid markers CD235 and CD71 (Figure 1C). Morphology showed that cells corresponded to orthochromatic erythroblasts (Figure 1C). Flow cytometry confirmed similar erythroid maturation markers between EMP3KO and WT lines (Figure 1D). In contrast to a previous study,^8^ no significant difference in proliferation was observed between the WT and EMP3KO iRBCs (Figure 1E), suggesting that EMP3 loss does not alter growth during primitive erythropoiesis.

During human embryogenesis, hematopoiesis proceeds through primitive and definitive developmental waves. The primitive wave produces red cells expressing embryonic ε‐ and ζ‐globins, while those from the definitive wave express fetal γ‐globin and later, adult β‐globin.^17^ Our iPSC differentiation recapitulates primitive hematopoiesis producing red cells similar to those observed in a 4–6 week embryo. To determine whether EMP3 loss could have distinct effects in hematopoietic developmental ontogeny, we examined EMP3 loss in definitive erythropoiesis by editing adult donor‐derived CD34+ HPCs using a Cas9:sgRNA RNP complex targeting *EMP3* exon 3.^18^ Cells edited with an *AAVS1* gRNA served as a control, as this site does not affect erythropoiesis.^18^ Untreated, control, and *EMP3*‐edited CD34+ cells were differentiated under erythroid conditions to generate definitive RBCs (Figure S2A). *EMP3*‐edited cells, assessed by PCR, Sanger sequencing, and Inference of CRISPR Edits analysis, decreased from 45% at day 7 to 20% by day 15 (Figure S2B), indicating no growth advantage of *EMP3*‐targeted cells. Erythroid expansion was comparable across conditions (Figure S2C), confirming that EMP3 may have minimal to no impact on red cell proliferation.

### 3.2 MAM‐null iRBCs as diagnostic tools

**FIGURE 2:**
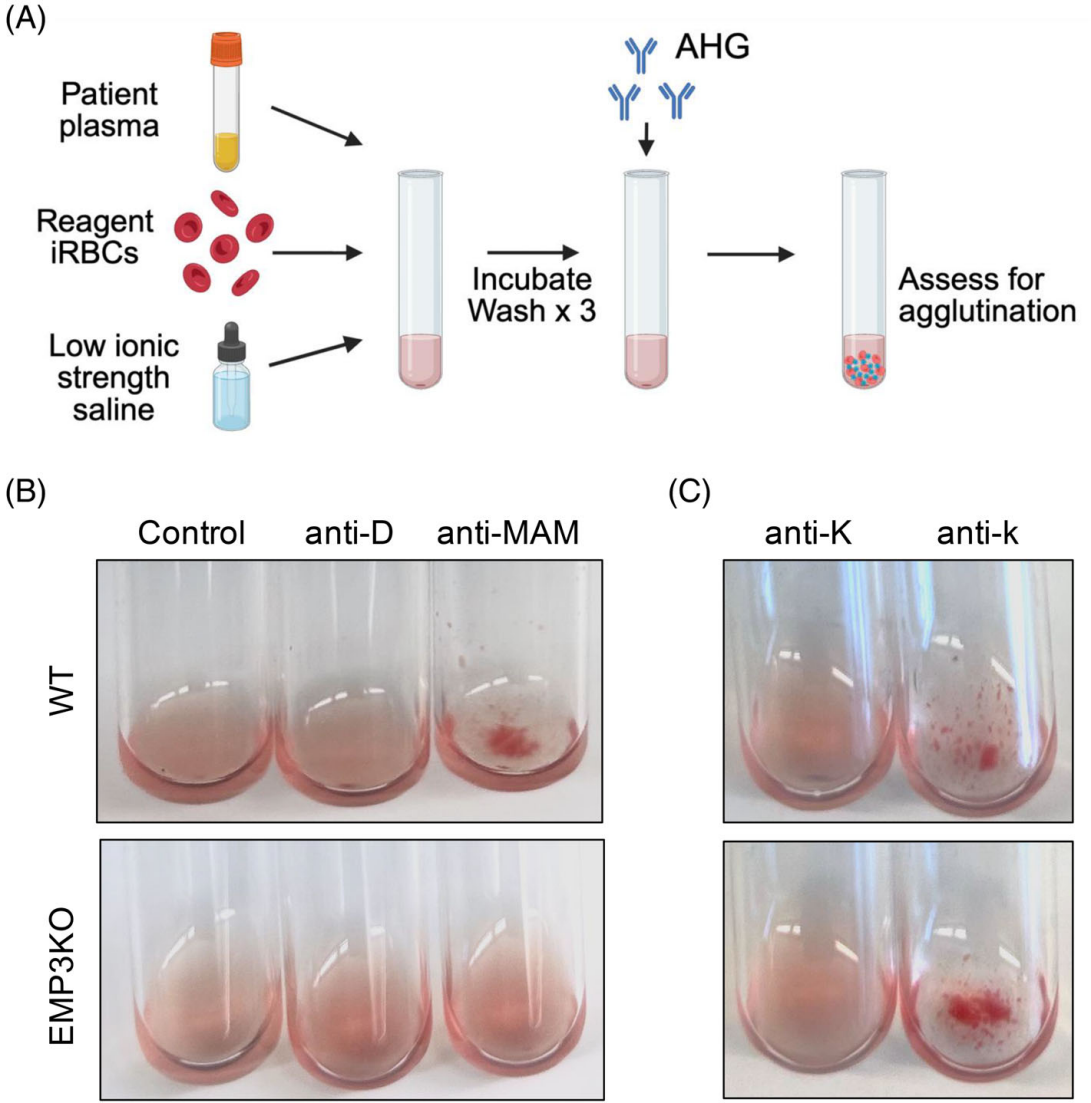
MAM‐negative iRBCs confirm anti‐MAM by tube agglutination assay. (A) Schematic of tube agglutination assay using patient plasma and reagent iRBCs. (B) Agglutination assays of WT and EMP3KO Rh null day 12 iRBCs using patient plasma containing no alloantibody (control), anti‐D, and anti‐MAM. (C) Agglutination assays with anti‐K and anti‐k monoclonal typing reagents consistent with the parent iPSC line genotype of K negative, k positive red cell phenotype. AHG, anti‐human globulin.

Agglutination assays are routinely used in blood banks to identify antibodies directed against RBC antigens (Figure 2A).^19^ Reagent cells that lack the specific antigen are essential for demonstrating a lack of agglutination to confirm the antibody specificity. Using WT and EMP3KO iRBCs, which are both Rh null, we tested agglutination using the gold standard tube method with plasma containing antibodies from RhD‐negative and two MAM‐negative individuals. Neither WT nor EMP3KO iRBCs reacted with anti‐D plasma, as expected since these cells are Rh null. WT iRBCs agglutinated in the presence of the anti‐MAM plasma from two different individuals, while EMP3KO iRBCs showed no agglutination (Figures 2B and S3). While primitive red cells express a subset of antigens of those on adult, definitive red cells,^10^, ^20^ EMP3KO iRBCs properly agglutinated with anti‐k reagent as these lines are K–k+ (Figure 2C). These findings confirm that EMP3KO iRBCs lack MAM antigen expression and demonstrate their utility as diagnostic reagent cells for antibody identification.

### 3.3 Transcriptomic assessment of EMP3 knockout iRBCs

**FIGURE 3:**
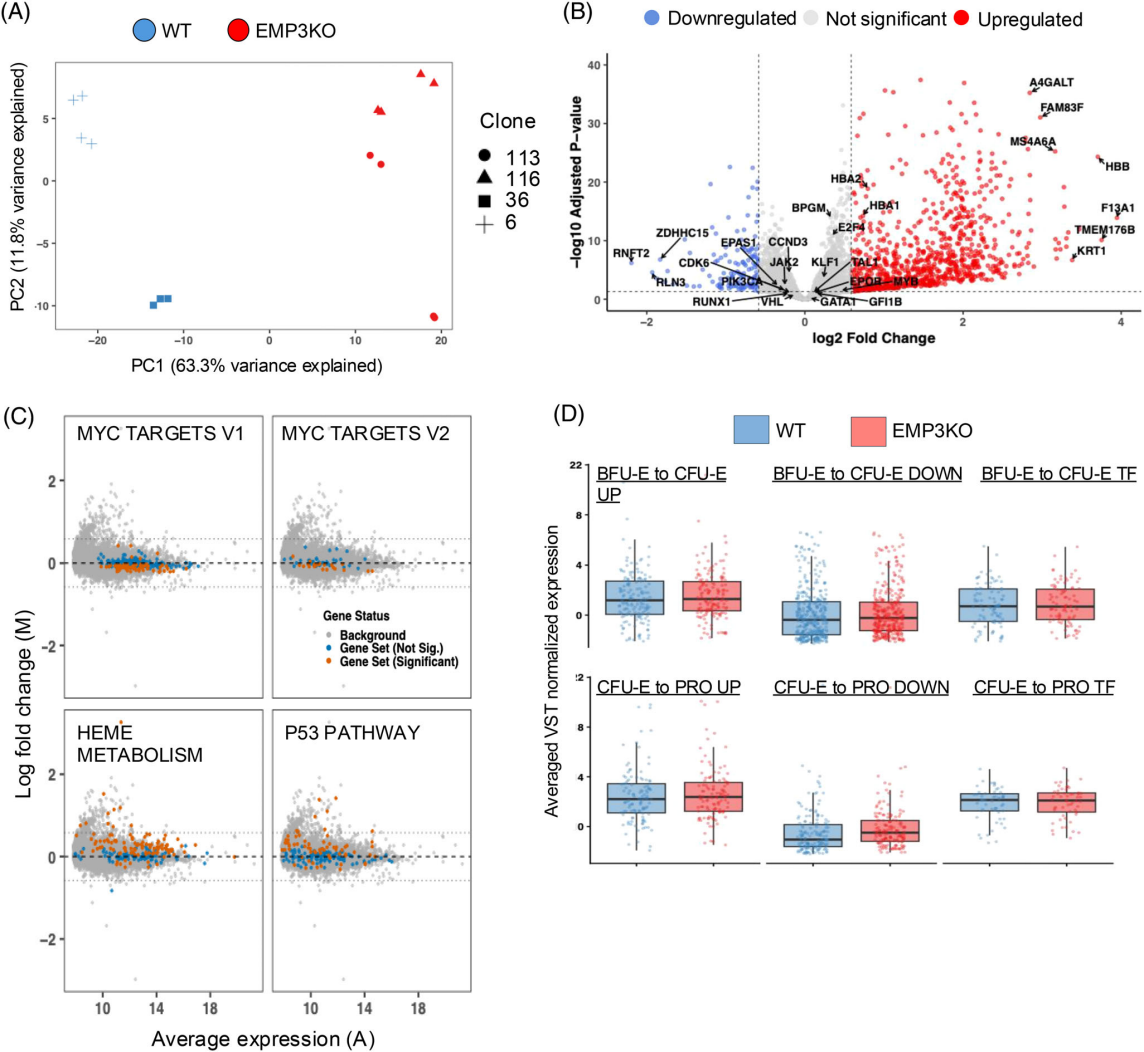
EMP3 knockout does not significantly affect erythroid differentiation and proliferation pathways. (A) Principal component analysis (PCA) of WT and EMP3KO day 12 iRBCs, derived from 2 iPSC clones per genotype (*n* = 7 biological replicates each). (B) Volcano plot showing differential gene expression including upregulated and downregulated genes in the EMP3KO line compared to WT. Erythroid‐specific genes (labeled) show no significant difference in expression between WT and EMP3KO. (C) MA plots showing the relationship between average gene expression (A; x‐axis) and log_2_ fold change (M; y‐axis) for four MSigDB Hallmark gene sets: MYC TARGETS V1, MYC TARGETS V2, HEME METABOLISM, and P53 PATHWAY. Genes belonging to each specified Hallmark set are highlighted in blue when not significantly differentially expressed and in orange when significantly differentially expressed (adjusted *p*‐value <0.05). Significance was assessed with DESeq2 using an adjusted *p*‐value cutoff of 0.05. All other genes are shown in gray. The horizontal dashed line denotes no change (M = 0), and the dotted lines mark a log_2_ fold change of ±0.58, corresponding to an approximate 1.5‐fold change in expression. (D) Genes identified by Li et al.^16^ to be upregulated (UP) and downregulated (DOWN) in the transition from erythroid burst forming unit (BFU‐E) to colony forming unit (CFU‐E) and from CFU‐E to proerythroblast (PRO), and transcription factors (TF) involved in these transitions showed no significant differences in erythroid differentiation at the transcriptional level with loss of EMP3. VST, variance stabilizing transformation.

Leveraging the genetic manipulability of iPSCs, precise knockout of a single erythroid antigen enables direct evaluation of its specific contribution to red cell development, maturation, or function. To assess the role of EMP3 in erythroblasts, we compared day 12 iRBCs from EMP3KO clones (113, 116) and Rh null WT clones (6, 36). Principal component analysis (PCA) revealed EMP3 genotype as the dominant source of transcriptomic variance, with PC1 accounting for 63.3% variance, while PC2 explained 11.8% (Figure 3A). Despite this global shift, differential gene expression (DGE) analysis showed no significant differences in core erythroid regulators or EPO–EPOR signaling components (Figure 3B), consistent with phenotypic and growth assays (Figure 1). Interestingly, globin genes such as *HBA1*, *HBA2*, and *HBB* were upregulated in the EMP3KO lines compared to WT, although HBB would not be expressed robustly in primitive stage erythroblasts (Figure 3B).

GSEA indicated positive enrichment of pathways such as IL6‐JAK‐STAT3 signaling, heme metabolism, and the p53 pathway in EMP3KO cells; MYC targets were negatively enriched (Figure S4). Although these pathways were enriched, changes in gene expression were marginal (<1.5‐fold), even among those that were statistically significant (*p*adj <0.05; Figure 3C). Additional examination of signatures linked to erythroid maturation from BFU‐E to CFU‐E, and to the proerythroblast stage identified by Li et al^16^ revealed no evidence of impaired differentiation at the transcriptomic level (Figure 3D). Overall, while EMP3 loss had a measurable transcriptomic shift, essential erythroid pathways remained intact, erythroid maturation markers were unaltered, and no proliferative advantage was observed, contrasting with previous non‐isogenic models.

## 4 DISCUSSION

We have generated a MAM‐null iPSC line that can be used to produce iRBCs for diagnostic testing. Our functional and transcriptomic analyses revealed no major differences in erythroid proliferation or differentiation with EMP3KO. These iRBCs were shown to be compatible with standard laboratory tube agglutination assays for the detection of plasma anti‐MAM antibodies, and in the future, may have therapeutic application.

The combination of iPSC technology and precise genetic engineering enables the creation of isogenic erythroid models in which a single antigen can be studied in isolation, advancing our understanding of red cell antigen biology and immune interactions. In contrast to a prior study, we did not observe a proliferative phenotype in RBCs differentiated from either EMP3KO iPSCs or *EMP3*‐edited adult CD34+ cells, which may reflect differences in genetic background. Thornton et al. utilized non‐isogenic donor‐derived CD34+ HPCs that were gender and age‐matched but genetically distinct from the primary MAM‐null cells, as they were obtained from different individuals.^8^ Of note, one of the control CD34+ HPCs, albeit unmatched for sex and age, proliferated similarly to one of the MAM‐negative CD34+ erythroid cultures. In contrast, our EMP3KO iRBCs were derived from isogenic parental cells (i.e., the same individual) and thus differing only at the targeted *EMP3* locus. While culture conditions may contribute to the differences in results between these studies, this is less likely given the overall similarity (Table S2).

EMP3KO iRBCs were tested against samples from two separate individuals who had MAM antibodies identified at a reference immunohematology laboratory and subsequently stored in their rare plasma collection. Anti‐MAM is uncommon, but likely underdiagnosed due to the scarcity of MAM‐negative reagent red cells needed to confirm the antibody. EMP3 knockout in iPSCs did not compromise erythroid differentiation or proliferation, supporting their use as specialized diagnostic red cells, particularly when donor cells with rare phenotypes are not readily available.

## FUNDING INFORMATION

This work was supported by the National Institutes of Health/National Heart Lung and Blood Institute U01 HL134696 (Stella T. Chou), HL169401 (Stella T. Chou), HL178144 (Stella T. Chou), and T32 HL007150, Mizuno Funds (Kaoru Takasaki), Children's Hospital of Philadelphia Breakthrough Funds (Stella T. Chou, Giulia Pavani), and a Distinguished Chair in Pediatrics (Stella T. Chou).

## CONFLICTS OF INTEREST STATEMENT

The authors declare no conflicts of interest.

## Supporting information


**Figure S1.** EMP3KO iPSC lines. Sequence alignment of complementary DNA made from RNA obtained from WT and EMP3KO day 12 iRBCs shows deletion of exon 3 in EMP3KO cells (dashed lines highlighted in red) as compared to the WT sequence. The amino acid sequence of the EMP3KO shows that exon 3 deletion leads to a premature stop codon in exon 4, which results in a truncated, nonfunctional EMP3 protein.

**Figure S2.** EMP3KO in adult peripheral CD34+ hematopoietic progenitor cells using RNP editing. (A) Representative flow cytometric analysis of cell surface erythroid maturation markers of day 12 untreated, *AAVS1* vector control and *EMP3*‐edited CD34+ cell‐derived RBCs. (B) Percent edited cells treated with *AAVS*1 or *EMP3* targeting vectors on days 7 (ns, *p* = 0.1022) and 15 (ns, *p* = 0.07) of erythroid culture. (C) Fold expansion of CD34+ cells treated with *AAVS1* or *EMP3* targeting vectors compared to untreated cells in erythroid culture on days 3, 6, 9, 12, and 15 (*n* = 3 independent assays).

**Figure S3.** MAM‐negative iRBCs identify anti‐MAM by tube agglutination assay with an independent anti‐MAM plasma sample. Agglutination assays of WT and EMP3KO Rh null day 12 iRBCs using plasma containing no RBC antibody (control), anti‐D, or anti‐MAM from a distinct individual from that shown in Figure 2.

**Figure S4.** MSigDB GSEA Hallmark pathways enriched in EMP3KO versus WT iRBCs. Significantly enriched MSigDB GSEA Hallmark pathways (*p*adj < 0.05) identified for differentially expressed genes (DEGs) between EMP3KO and WT (baseline). Pathways with a positive Normalized Enrichment Score (NES) are enriched with upregulated genes in EMP3KO, whereas pathways with a negative NES are enriched with downregulated genes in EMP3KO.

**Table S1.** gDNA and PCR primer sequences for iPSC editing and CD34 RNP editing.
**Table S2.** Comparison of CD34+ HPC erythroid culture conditions to Thornton et al cultures^13^ Thornton et al. utilized two distinct culture systems for erythroid differentiation of CD34+ HPCs from blood derived from MAM‐negative and control individuals; the International Blood Group Reference Laboratory (IBGRL) protocol, based on Griffiths et al.^14^ and the Lund University protocol was adapted from Giarratana et al.^15^ and Flygare et al.^16^ Gunawardena et al. indicates culture conditions for differentiation of both primary adult‐derived HPCs and iPSC‐derived HPCs in erythroid lineage in this study. Pen‐strep, penicillin‐streptomycin; SFEM, serum‐free expansion media (STEMCELL Technologies), SCF, stem cell factor; IL‐3, interleukin‐3; Epo, erythropoietin; Iron sat transferrin, iron‐saturated transferrin; TPO, thrombopoieitin; FLT‐3 ligand, FMS‐like tyrosine kinase 3 ligand; dex, dexamethasone; FBS, fetal bovine serum; Holo‐T, holo‐transferrin.

## Data Availability

The data that support the findings of this study are available from the corresponding author upon reasonable request.
