# Supplementary figures and images for: Rare antigen‐negative red blood cells from pluripotent stem cells for precision transfusion medicine
Source: Transfusion. 2026 Apr 24;66(7):1257–64. doi: 10.1111/trf.70243 (PMC13350276; doi:10.1111/trf.70243)

EMP3 cDNA

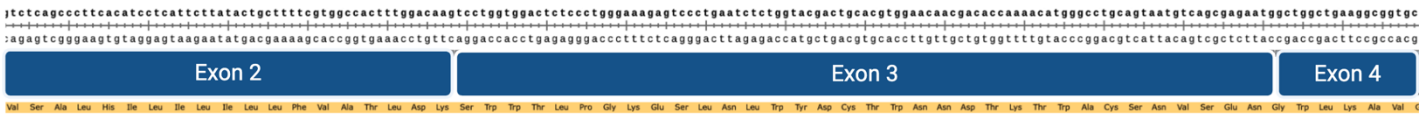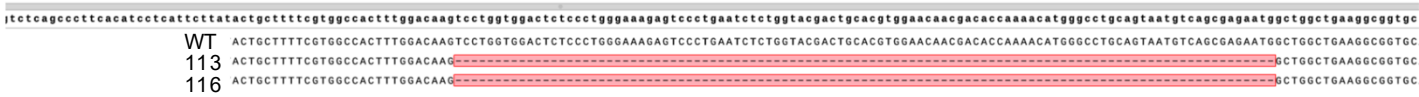

EMP3KO amino acid sequence

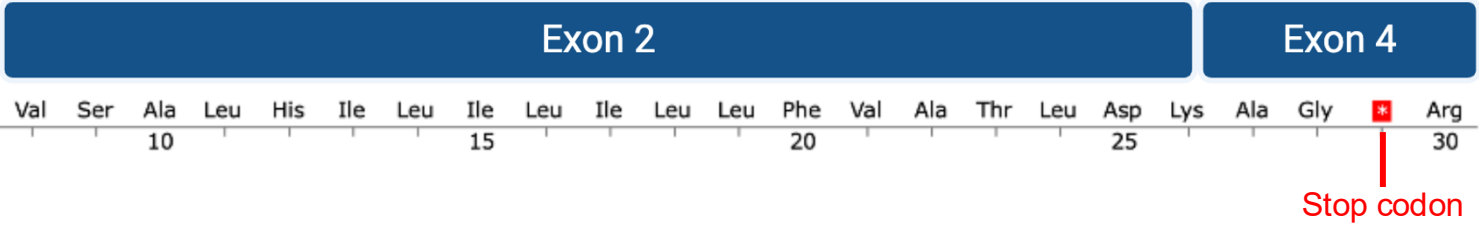

Figure S1

Supplement: Supplementary file 1 — Figure S1. EMP3KO iPSC lines. Sequence alignment of complementary DNA made from RNA obtained from WT and EMP3KO day 12 iRBCs shows deletion of exon 3 in EMP3KO cells (dashed lines highlighted in red) as compared to the WT sequence. The amino acid sequence of the EMP3KO shows that exon 3 deletion leads to a premature stop codon in exon 4, which results in a truncated, nonfunctional EMP3 protein. [file TRF-66-1257-s003.pdf]

A

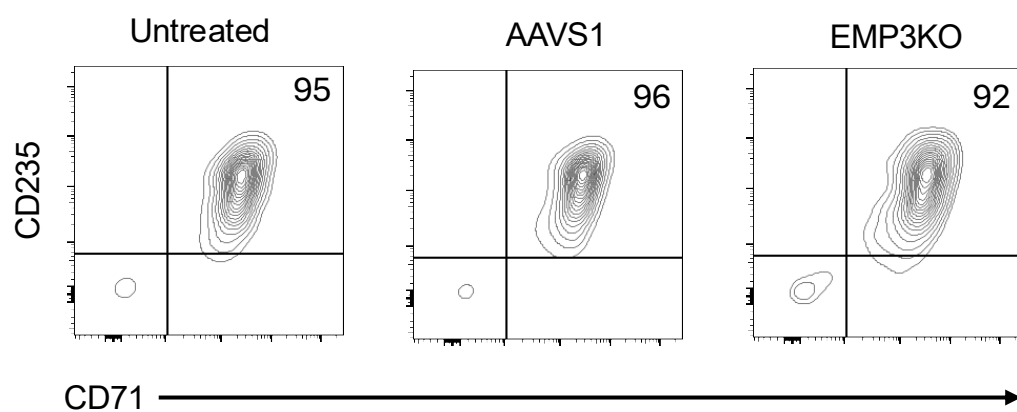

B

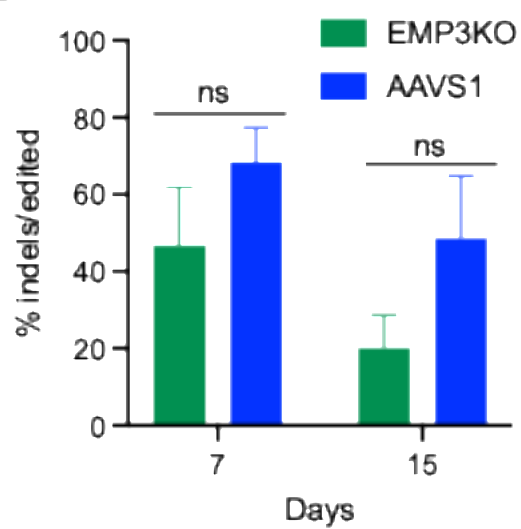

C

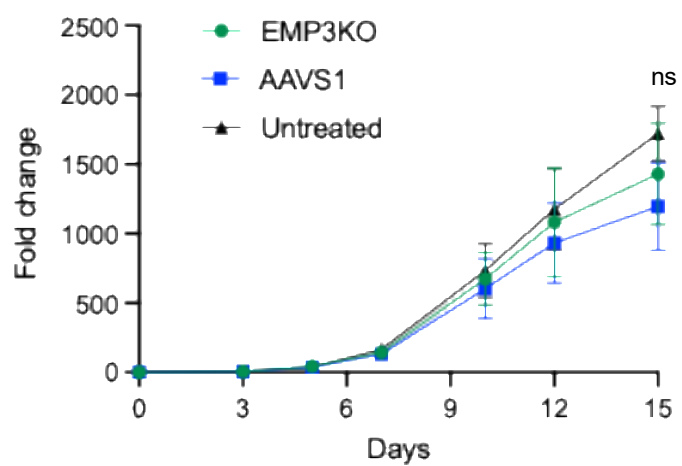

Figure S2

Supplement: Supplementary file 2 — Figure S2. EMP3KO in adult peripheral CD34+ hematopoietic progenitor cells using RNP editing. (A) Representative flow cytometric analysis of cell surface erythroid maturation markers of day 12 untreated, AAVS1 vector control and EMP3‐edited CD34+ cell‐derived RBCs. (B) Percent edited cells treated with AAVS1 or EMP3 targeting vectors on days 7 (ns, p = 0.1022) and 15 (ns, p = 0.07) of erythroid culture. (C) Fold expansion of CD34+ cells treated with AAVS1 or EMP3 targeting vectors compared to untreated cells in erythroid culture on days 3, 6, 9, 12, and 15 (n = 3 independent assays). [file TRF-66-1257-s004.pdf]

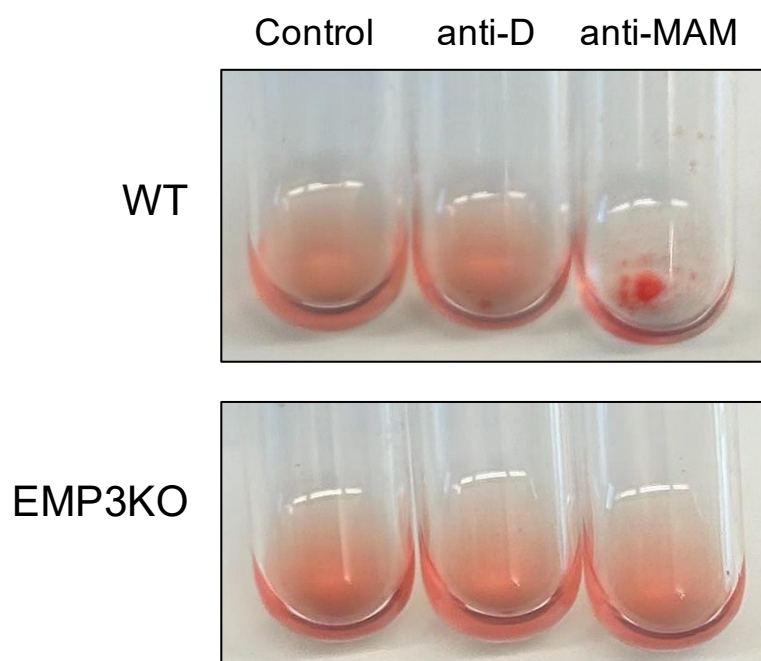

Figure S3

Supplement: Supplementary file 3 — Figure S3. MAM‐negative iRBCs identify anti‐MAM by tube agglutination assay with an independent anti‐MAM plasma sample. Agglutination assays of WT and EMP3KO Rh null day 12 iRBCs using plasma containing no RBC antibody (control), anti‐D, or anti‐MAM from a distinct individual from that shown in Figure 2. [file TRF-66-1257-s002.pdf]

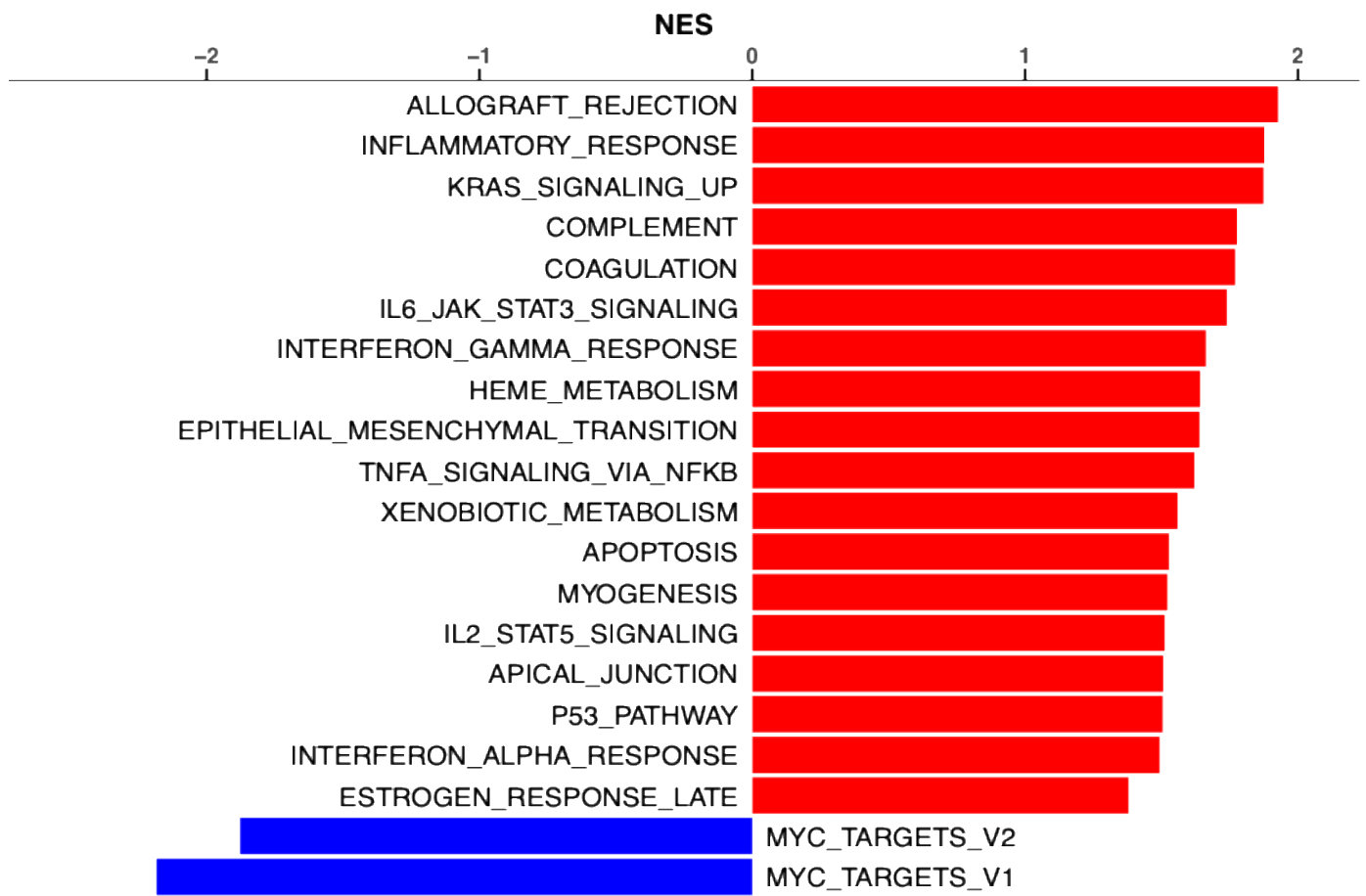

Figure S4

Supplement: Supplementary file 4 — Figure S4. MSigDB GSEA Hallmark pathways enriched in EMP3KO versus WT iRBCs. Significantly enriched MSigDB GSEA Hallmark pathways (padj < 0.05) identified for differentially expressed genes (DEGs) between EMP3KO and WT (baseline). Pathways with a positive Normalized Enrichment Score (NES) are enriched with upregulated genes in EMP3KO, whereas pathways with a negative NES are enriched with downregulated genes in EMP3KO. [file TRF-66-1257-s005.pdf]
