# Supplementary material for: Rare antigen‐negative red blood cells from pluripotent stem cells for precision transfusion medicine
Source: Transfusion. 2026 Apr 24;66(7):1257–64. doi: 10.1111/trf.70243 (PMC13350276; doi:10.1111/trf.70243)
Supplement: Supplementary file 5 — Table S1. gDNA and PCR primer sequences for iPSC editing and CD34 RNP editing. Table S2. Comparison of CD34+ HPC erythroid culture conditions to Thornton et al cultures13 Thornton et al. utilized two distinct culture systems for erythroid differentiation of CD34+ HPCs from blood derived from MAM‐negative and control individuals; the International Blood Group Reference Laboratory (IBGRL) protocol, based on Griffiths et al.14 and the Lund University protocol was adapted from Giarratana et al.15 and Flygare et al.16 Gunawardena et al. indicates culture conditions for differentiation of both primary adult‐derived HPCs and iPSC‐derived HPCs in erythroid lineage in this study. Pen‐strep, penicillin‐streptomycin; SFEM, serum‐free expansion media (STEMCELL Technologies), SCF, stem cell factor; IL‐3, interleukin‐3; Epo, erythropoietin; Iron sat transferrin, iron‐saturated transferrin; TPO, thrombopoieitin; FLT‐3 ligand, FMS‐like tyrosine kinase 3 ligand; dex, dexamethasone; FBS, fetal bovine serum; Holo‐T, holo‐transferrin. [file TRF-66-1257-s001.pdf]

Supplemental Information

Supplemental Methods

iPSC-derived hematopoietic progenitor cell (HPC) erythroid differentiation

On day 8 of embryoid body culture, HPCs were harvested and cultured in erythroid medium for 12 days. Erythroid medium consisted of Iscove's Modified Dulbecco's Media (IMDM, Corning), 5% Octaplas (Octapharma), 1% penicillin-streptomycin (Corning), 1 mg/mL holo-transferrin (Sigma), 10 µg/mL insulin (Sigma), and 3 U/mL heparin (Sigma). Cultures were supplemented with 3 U/mL Erythropoietin (Epo, Amgen) and 10 ng/µL stem cell factor (SCF, R&D Systems) on days 0-5, and 3 U/mL Epo alone on days 6-12. On day 0 of erythroid differentiation, cells were seeded at 1 x 10^5 cells/mL. Fresh medium was added every 3 days to target a density of ~1 x 10^6 cells/mL, with cytokines replenished to maintain final concentrations.[1]

Adult CD34+ hematopoietic progenitor cell ribonucleoprotein editing and erythroid culture

Mobilized human peripheral blood $CD34^+$ cells (Fred Hutchinson Center) were gene edited using ribonucleoprotein (RNP) editing with gRNAs targeting EMP3 or AAVS1 following an established protocol.[2] gRNA and primer sequences are in **Table S1**. Cells were cultured for 48 hours in SFEMII medium (Stem Cell Technologies) containing recombinant human SCF 100 ng/ml, Flt3 ligand 100 ng/ml, Thrombopoietin 100 ng/ml and interleukin-3 20 ng/ml (R&D Systems).[2] Single guide RNA targeting EMP3 or AAVS1[2] were prepared according to the manufacturer's instructions, and ribonucleoprotein (RNP) complexes were assembled with 30 pmol of spCas9 (1:1.5 Cas9:sgRNA molar ratio). $CD34^+$ cells (2.5 x10^5 per condition) were transfected with RNP complexes using the P3 Primary Cell 4D-Nucleofector kit and CA137 program (Lonza), then placed into erythroid differentiation medium and cultured for 18 days. For days 0 – 12 of erythroid culture (phases 1 and 2), fresh media was added every 2-3 days to keep cell concentration at 1 – 2 x 10^5 cells/ml. Starting on day 12 of culture until end of culture on day 18, cells were kept at 1 x 10^6 cells/ml. Cytokines were replenished with every feed to maintain final concentrations. Phase

1 medium (days 0 – 7 of erythroid culture) consisted of base IMDM supplemented with 5% Octaplas, 1% penicillin-streptomycin, 3 U/mL heparin, 10 ug/ml insulin, 3 U/ml Epo, 200 ug/ml holo-transferrin, 10 ng/ml SCF, and 1 ng/ml IL-3. Phase 2 medium (days 7 – 12) consisted of base IMDM supplemented with 5% Octaplas, 1% penicillin-streptomycin, 3 U/mL heparin, 10 ug/ml insulin, 3 U/ml Epo, 200 ug/ml holo-transferrin and 10 ng/ml SCF. Phase 3 medium (days 12 – 18) consisted of IMDM supplemented with 5% Octaplas, 1% penicillin-streptomycin, 3 U/mL heparin, 10 ug/ml insulin, 3 U/ml Epo and 1 mg/ml holo-transferrin. Editing efficiency was assessed at days 7 and 15 of culture. Genomic DNA was isolated at multiple time points with QuickExtract™ DNA Extraction Solution (Lucigen). A 1:3 dilution of genomic DNA was used as a template to amplify the gRNA target region with KAPA2G Fast ReadyMix (Roche). PCR products were analyzed by Sanger sequencing (Genewiz), and insertion-deletion (InDel) frequencies were quantified using Synthego Inference of CRISPR Edits (ICE) software.

Flow cytometry analysis

Flow cytometry analysis was performed on FACS Canto II (BD Biosciences) or FACSymphony (BD). Antibodies used for surface marker staining are shown in **Table S3**. Cells were incubated for 20 minutes in antibody diluted in FACS buffer. After incubation, cells were washed and then fixed in 1% paraformaldehyde. Flow cytometry data were analyzed using FlowJo 10.10.0 (Becton Dickinson & Company).

Morphologic analysis

350,000 day 12 iRBCs were spun onto a microscope slide using a cytocentrifuge at 200 rpm for one minute. The slide was then stained with May Grünwald stain for two minutes followed by Giemsa stain for ten minutes, after which it was washed in deionized water and allowed to air dry prior to application of a glass coverslip. Slides were imaged on an Olympus BX60 with a 20X objective.

Supplemental Information

Statistical analysis

Data were analyzed by 2-tailed, unpaired *t*-test for comparison of 2 conditions or ordinary one-way ANOVA for multiple comparisons. All data are summarized as the mean ± standard error of the mean (SEM) with statistically significant differences indicated with corresponding *p*-values (*$p < 0.05$, nonsignificant if $p > 0.05$).

Bulk RNA sequence analysis

RNA was collected from day 12 iRBCs from WT iPSC clones 6 and 36 and EMP3KO clones 113 and 116 (n = 3 differentiations). Total RNA was extracted from cells using the RNeasy Mini Kit (Qiagen) according to the manufacturer's instructions. RNA quality and integrity were measured by spectrophotometer (Nanodrop, ThermoFisher Scientific) and capillary electrophoresis separation (LabChip GX Bioanalyzer, Perkin Elmer). Library preparation (NebNext Stranded mRNA with poly-A enrichment, Illumina), and sequencing (NextSeq 1000/2000, Illumina) were performed by the High Throughput Sequencing Core (CHOP). Read quality was assessed with FastQC (v0.11.9).[3] Adapter sequences and low-quality bases were trimmed using Trim Galore (v0.6.7).[4] High-quality reads were aligned to the human GRCh38 reference genome using STAR (v2.7.10a)().[5] Gene-level abundances were estimated with RSEM (v1.3.1)[6] using Ensembl comprehensive gene annotations.

RSEM gene-level counts were imported into the gene expression differential analysis workflow using tximport[7], and low-count genes were pre-filtered to retain only those with at least 10 counts in at least 7 samples (the smallest biological group size). Data normalization was performed using a Variance Stabilizing Transformation (VST) implemented in DESeq2 (v1.38.3)[8] to facilitate quality-control and read-level comparative analyses. Differential gene expression (DGE) was performed with DESeq2 using a negative binomial generalized linear model testing the effect of genotype. Pairwise contrasts were defined to compare EMP3KO versus WT iRBCs, $\log_2$ fold-change estimates were shrunk using the ashr adaptive shrinkage

estimator[9] to improve effect-size estimates for low-count genes, and differentially expressed genes were defined as those with adjusted p-value (padj) < 0.05.

Gene set enrichment analysis (GSEA) was performed using the fgsea package[10], using the entire DGE result set pre-ranked by $\log_2$ fold change as input. The analysis utilized MSigDB Hallmark pathways (category H)[11]. MA plots were generated to show the individual gene expression differences (M) relative to their average abundance (A) for quality control (**Figure 3C**). Pathways positively (red) and negatively (blue) enriched in EMP3KO cells compared to WT are shown in **Figure S4**. Separate analyses were conducted to examine specific subsets of genes related to erythroid differentiation, including gene sets identified by Li et al[12] that are up- or downregulated during the transition from erythroid burst forming unit (BFU-E) to colony forming unit (CFU-E) and from CFU-E to proerythroblast, along with known transcription factors involved in these transitions **(Figure 3D)**.

Supplemental Information

Table S1. gDNA and PCR primer sequences for iPSC editing and CD34 RNP editing

Targeting strategy	gRNA sequence	PCR primer sequence
iPSC CRISPR-Cas9 gene editing	*EMP3* intron 2: GCGTGGCAAGATTCCAAAG<br>*EMP3* intron 3: CCCACTCCAACTTTGTTGG	*EMP3* intron 1 (forward) primer: CCAGCGCGAATCCTATCCAT<br>*EMP3* intron 3 (reverse) primer: ACAGGCGGGACATGCATTAG
CD34+ HPC RNP editing	*EMP3:*<br>UUUCCCAGGGAGAGUCCACC<br>*AAVS1:*<br>GUCCCCUCCACCCCACAGUG	*EMP3* exon 3 (forward):<br>GGGATTACAGGCATGAACCA<br>*EMP3* exon 3 (reverse):<br>AGAGGTAGGTACATCCAGACAG<br>*AAVS1* (forward):<br>CAGCTCAGGTTCTGGGAGAG<br>*AAVS1* (reverse):<br>CTTGTAGGCCTGCATCATCA

Supplemental Information

Table S2. Comparison of CD34+ HPC erythroid culture conditions to Thornton et al cultures[13]
Thornton et al utilized two distinct culture systems for erythroid differentiation of CD34+ HPCs from blood derived from MAM-negative and control individuals; the International Blood Group Reference Laboratory (IBGRL) protocol, based on Griffiths et al[14] and the Lund University protocol was adapted from Giarratana et al[15] and Flygare et al.[16] Gunawardena et al indicates culture conditions for differentiation of both primary adult-derived HPCs and iPSC-derived HPCs in erythroid lineage in this study. Pen-strep, penicillin-streptomycin; SFEM, serum-free expansion media (STEMCELL Technologies), SCF, stem cell factor; IL-3, interleukin-3; Epo, erythropoietin; Iron sat transferrin, iron-saturated transferrin; TPO, thrombopoieitin; FLT-3 ligand, FMS-like tyrosine kinase 3 ligand; dex, dexamethasone; FBS, fetal bovine serum; Holo-T, holo-transferrin.

	**Thornton et al:**<br>IBGRL[14]	**Thornton et al:**<br>Lund University[15, 16]	**Gunawardena et al**<br>CD34+ adult primary HSPCs	**Gunawardena et al**<br>iPSC-derived HPCs
**Culture duration**	21 days	18 days	18 days	12 days
**Cell density**	Initial: 0.5-1 x 10^5 cells/ml<br>At 100 ml, transferred into stirred glass spinner vessels and maintained at 1-6 x 10^6 cells/ml	1-2 x 10^5 cells/ml	<u>Days 1-12:</u><br>1-2 x 10^5 cells/ml<br><u>Days 12-18:</u><br>1 x 10^6 cells/ml	Initial:<br>1 x 10^5 cells/ml<br><u>Days 3, 6, 9:</u><br>Maintain at 1 x 10^6 cells/ml
**Base media**	IMDM<br>AB serum 3%<br>Serum albumin 2 mg/ml<br>Heparin 3 U/ml<br>Insulin 10 ug/ml<br>Transferrin 200 ug/ml	SFEM	IMDM<br>Octaplas 5%<br>Pen-strep 1%<br>Heparin 3 U/ml<br>Insulin 10 ug/ml	IMDM<br>Octaplas 5%<br>Pen-strep 1%<br>Heparin 3 U/ml<br>Insulin 10 ug/ml
**First stage**	<u>Days 0-11:</u><br>SCF 10 ug/ml<br>IL-3 1 ug/ml<br>Epo 3 U/ml	<u>Days 0-7:</u><br>SCF 50 ng/ml<br>TPO 50 ng/ml<br>FLT-3 ligand 50 ng/ml<br>IL-3 5 ng/ml<br>Dex 100 nmol/L	<u>Days 0-7:</u><br>SCF 10 ng/ml<br>IL-3 1 ng/ml<br>Epo 3 U/ml<br>Holo-T 200 ug/ml	--
**Second stage**	<u>Days 11-14:</u><br>SCF 10 ug/ml<br>Epo 3 U/ml<br>Iron sat transferrin 800 ug/ml	<u>Days 7-14:</u><br>SCF 50 ng/ml<br>Epo 2 U/ml<br>Dex 100 nmol/L	<u>Days 7-12:</u><br>SCF 10 ng/ml<br>Epo 3 U/ml<br>Holo-T 200 ug/ml	<u>Days 0-6:</u><br>SCF 10 ng/ul<br>Epo 3 U/ml<br>Holo-T 1 mg/ml
**Third stage**	<u>Days 14-21:</u><br>Epo 3 U/ml<br>Iron sat transferrin 800 ug/ml	<u>Days 14-18:</u><br>FBS 30%<br>Epo 3 U/ml<br>Holo-T 300 ug/ml	<u>Days 12-18:</u><br>Epo 3 U/ml<br>Holo-T 1 mg/ml	<u>Days 6-12:</u><br>Epo 3 U/ml<br>Holo-T 1 mg/ml

Supplemental Information

Table S3. Fluorophore-conjugated antibodies against cell surface markers used for flow cytometric analysis

**Cell surface marker**	**Fluorophore**	**Company**	**Dilution**
CD235	APC	BD Pharmingen<br>Cat: 551336<br>Lot: 4253034	1:5000
CD235	FITC	BD Pharmingen<br>Cat: 561017<br>Lot: 6051562	1:5000
CD71	FITC	BD Pharmingen<br>Cat: 555536<br>Lot: 4031567	1:50
CD36	APC	BioLegend<br>Cat: 336208<br>Lot: B462702	1:100
CD44	PE	BioLegend<br>Cat: 103024<br>Lot: B115361	1:100
CD41	FITC	BioLegend<br>Cat: 303704<br>Lot B458671	1:40
CD45	APC	BD Biosciences<br>Cat: 555485<br>Lot: 4309589	1:20
CD18	FITC	BD Pharmingen<br>Cat: 555923<br>Lot: 5171842	1:20
CD49d	APC	R&D Systems<br>Cat: FAB1354R<br>Lot: 1656315	1:100
Band3	FITC	Courtesy of Xiuli An, New York Blood Center	1:100

Supplemental Information

Supplemental Figure Legends

Figure S1. EMP3KO iPSC lines. Sequence alignment of complementary DNA made from RNA obtained from WT and EMP3KO day 12 iRBCs shows deletion of exon 3 in EMP3KO cells (dashed lines highlighted in red) as compared to the WT sequence. The amino acid sequence of the EMP3KO shows that exon 3 deletion leads to a premature stop codon in exon 4, which results in a truncated, nonfunctional EMP3 protein.

Figure S2. EMP3KO in adult peripheral CD34+ hematopoietic progenitor cells using RNP editing. A) Representative flow cytometric analysis of cell surface erythroid maturation markers of day 12 untreated, *AAVS1* vector control and *EMP3*-edited CD34+ cell-derived RBCs. **B)** Percent edited cells treated with *AAVS*1 or *EMP3* targeting vectors on days 7 (ns, p = 0.1022) and 15 (ns, p = 0.07) of erythroid culture. **C)** Fold expansion of CD34+ cells treated with *AAVS1* or *EMP3* targeting vectors compared to untreated cells in erythroid culture on days 3, 6, 9, 12, and 15 (n = 3 independent assays).

Figure S3. MAM-negative iRBCs identify anti-MAM by tube agglutination assay with an independent anti-MAM plasma sample. Agglutination assays of WT and EMP3KO Rh null day 12 iRBCs using plasma containing no RBC antibody (control), anti-D, or anti-MAM from a distinct individual from that shown in Figure 2.

Figure S4. MSigDB GSEA Hallmark pathways enriched in EMP3KO versus WT iRBCs. Significantly enriched MSigDB GSEA Hallmark pathways (padj < 0.05) identified for differentially expressed genes (DEGs) between EMP3KO and WT (baseline). Pathways with a positive Normalized Enrichment Score (NES) are enriched with upregulated genes in EMP3KO, whereas pathways with a negative NES are enriched with downregulated genes in EMP3KO.

Supplemental Information

References

1. An HH, Gagne AL, Maguire JA, Pavani G, Abdulmalik O, Gadue P, French DL, Westhoff CM, Chou ST. The use of pluripotent stem cells to generate diagnostic tools for transfusion medicine. *Blood.* 2022;140(15):1723-1734.
2. Pavani G, Laurent M, Fabiano A, Cantelli E, Sakkal A, Corre G, Lenting PJ, Concordet J-P, Toueille M, Miccio A, Amendola M. Ex vivo editing of human hematopoietic stem cells for erythroid expression of therapeutic proteins. *Nature Communications.* 2020;11(1):3778.
3. S. A. FastQC: A quality control tool for high throughput sequence data. . https://www.bioinformatics.babraham.ac.uk/projects/fastqc/.
4. F K. Trim Galore! Babraham Bioinformatics. . https://www.bioinformatics.babraham.ac.uk/projects/trim_galore/.
5. Dobin A, Davis CA, Schlesinger F, Drenkow J, Zaleski C, Jha S, Batut P, Chaisson M, Gingeras TR. STAR: ultrafast universal RNA-seq aligner. *Bioinformatics.* 2013;29(1):15-21.
6. Li B, Dewey CN. RSEM: accurate transcript quantification from RNA-Seq data with or without a reference genome. *BMC Bioinformatics.* 2011;12:323.
7. Soneson C, Love MI, Robinson MD. Differential analyses for RNA-seq: transcript-level estimates improve gene-level inferences. *F1000Res.* 2015;4:1521.
8. Love MI, Huber W, Anders S. Moderated estimation of fold change and dispersion for RNA-seq data with DESeq2. *Genome Biol.* 2014;15(12):550.
9. Stephens M. False discovery rates: a new deal. *Biostatistics.* 2017;18(2):275-294.
10. Korotkevich G SV, Sergushichev A. . Fast gene set enrichment analysis.
11. Liberzon A, Birger C, Thorvaldsdottir H, Ghandi M, Mesirov JP, Tamayo P. The Molecular Signatures Database (MSigDB) hallmark gene set collection. *Cell Syst.* 2015;1(6):417-425.
12. Li J, Hale J, Bhagia P, Xue F, Chen L, Jaffray J, Yan H, Lane J, Gallagher PG, Mohandas N, Liu J, An X. Isolation and transcriptome analyses of human erythroid progenitors: BFU-E and CFU-E. *Blood.* 2014;124(24):3636-3645.
13. Thornton N CV, Tilley L, Green CA, Tay CL, Griffiths RE, Singleton BK, Spring F, Walser P, Alattar AG, Jones B, Laundy R, Storry JR, Möller M, Wall L, Charlewood R, Westhoff CM, Lomas-Francis C, Yahalom V, Feick U, Seltsam A, Mayer B, Olsson ML, Anstee DJ. Disruption of the tumour-associated EMP3 enhances erythroid proliferation and causes the MAM-negative phenotype. *Nature Communications.* 2020;11(1):3569.
14. Griffiths RE, Kupzig S, Cogan N, Mankelow TJ, Betin VM, Trakarnsanga K, Massey EJ, Lane JD, Parsons SF, Anstee DJ. Maturing reticulocytes internalize plasma membrane in glycophorin A-containing vesicles that fuse with autophagosomes before exocytosis. *Blood.* 2012;119(26):6296-6306.
15. Giarratana MC, Kobari L, Lapillonne H, Chalmers D, Kiger L, Cynober T, Marden MC, Wajcman H, Douay L. Ex vivo generation of fully mature human red blood cells from hematopoietic stem cells. *Nat Biotechnol.* 2005;23(1):69-74.
16. Flygare J, Rayon Estrada V, Shin C, Gupta S, Lodish HF. HIF1alpha synergizes with glucocorticoids to promote BFU-E progenitor self-renewal. *Blood.* 2011;117(12):3435-3444.
